# No Gender Differences in Egocentric and Allocentric Environmental Transformation After Compensating for Male Advantage by Manipulating Familiarity

**DOI:** 10.3389/fnins.2018.00204

**Published:** 2018-03-28

**Authors:** Raffaella Nori, Laura Piccardi, Andrea Maialetti, Mirco Goro, Andrea Rossetti, Ornella Argento, Cecilia Guariglia

**Affiliations:** ^1^Department of Psychology, University of Bologna, Bologna, Italy; ^2^Life, Health and Environmental Science Department L'Aquila University, L'Aquila, Italy; ^3^Neuropsychology Unit, IRCCS Santa Lucia Foundation, Rome, Italy; ^4^Department of Psychology, University of Rome, Rome, Italy

**Keywords:** gender differences, allocentric frames of reference, egocentric frames of reference, change of perspective, learning time

## Abstract

The present study has two-fold aims: to investigate whether gender differences persist even when more time is given to acquire spatial information; to assess the gender effect when the retrieval phase requires recalling the pathway from the same or a different reference perspective (egocentric or allocentric). Specifically, we analyse the performance of men and women while learning a path from a map or by observing an experimenter in a real environment. We then asked them to reproduce the learned path using the same reference system (map learning vs. map retrieval or real environment learning vs. real environment retrieval) or using a different reference system (map learning vs. real environment retrieval or vice versa). The results showed that gender differences were not present in the retrieval phase when women have the necessary time to acquire spatial information. Moreover, using the egocentric coordinates (both in the learning and retrieval phase) proved easier than the other conditions, whereas learning through allocentric coordinates and then retrieving the environmental information using egocentric coordinates proved to be the most difficult. Results showed that by manipulating familiarity, gender differences disappear, or are attenuated in all conditions.

## Introduction

Human beings orient themselves through the environment by using different strategies to represent the space. The notion of “frame of reference” refers to the way in which individuals represent landmarks and their spatial location as well as their own position with respect to the environmental objects.

In spatial cognition, individuals may use two different spatial frames of references: “egocentric” (body-centered) and “allocentric” (world-centered) (Burgess, 2006, 2008; Arleo and Rondi-Reig, 2007). Specifically, an individual may locate environmental features by (a) referring to his own position, namely an egocentric frame of reference or (b) referring to the spatial and configurational properties of environmental features, namely an allocentric frame of reference (Galati et al., 2000; for a review see Avraamides and Kelly, 2008).

For example, when individuals actually navigate through an environment they memorize spatial locations considering them with respect to their position, whereas when they plan or study a path by observing it on a map they represent the space regardless of their own position. Both humans and animals form an environmental representation by integrating internal cues (e.g., self-motion; proprioceptive information; visual cues) with external ones (e.g., the relationships between environmental landmarks; positional and directional environmental cues: Jacobs and Schenk, 2003; Cheng, 1986). Therefore, the “map-like” environmental representation depends on egocentric and allocentric reference frames and the continuous translation of information coming from these two systems.

According to Wolbers and Hegarty (2010), spatial navigation requires three essential elements: (1) Spatial cues, (2) Computational mechanisms, and (3) Spatial representations. Spatial cues have a role in extracting information about our own position in the environment. They might refer to either environmental cues (i.e., such as landmarks, geometric structure of the environment) or self-motion cues (i.e., such as proprioceptive, vestibular, and motion cues). Computational mechanisms include both spatial computations and more general executive processes. On the one hand, spatial computations include space perception, translating between egocentric and allocentric frames of reference, computing distance and directions toward an invisible goal, imagining shifts in spatial perspective. On the other hand, executive processes involve novelty detection, selection, and maintenance of the navigational goal, route planning, and conflict resolution. Spatial representations include both offline and online spatial representations. Most of the models of human spatial navigation focused on the attributes of the spatial representations, that is the way in which spatial knowledge is organized.

As pointed out by Mou et al. (2006), a path requires the computation of a precise self-to-object spatial relation to guide locomotion, while planning a route to a distant goal and maintaining a sense of direction in a large-scale environment requires object-to-object spatial relation. The egocentric frame of reference considers the body as the center of the environmental organization so the spatial mental representation is biased by the relation between the body's position and the spatial location. On the contrary, the allocentric frame of reference is specified independently of the body's position (e.g., Waller et al., 2002). As a consequence, during navigation, individuals process egocentric and allocentric environmental cues that have a role in building and retrieving topographic long-term memory. Contemporary models of human spatial memory and navigation attempt to specify the role of both egocentric and allocentric information. Wang and Spelke, (2000, 2002) suggested that spatial memory was solely supported by egocentric references. Specifically, they suggested the existence of two stages, namely an egocentric process and a geometric module. The former involves a viewpoint-dependent scene recognition and a spatial updating of location by self-motion information whereas the latter has no role in representing locations but only in representing the geometry of the environment in spite of the individual's position. From this point of view, an allocentric frame of reference is not openly considered, if not with the purpose to support reorientation when the path integration system (i.e., the capacity to use cues generated by one's own movements to update one's position in the environment) breaks down. However, experimental data indicate a “two-system” model of parallel egocentric and allocentric representations in memory (e.g., Burgess et al., 2001; Burgess, 2006; Waller and Hodgson, 2006). Mou et al. (2004) proposed another model of spatial memory and navigation including an egocentric and an environmental subsystem. The former computes and represents the transient self-to-object spatial relations needed for locomotion, and decays rapidly in the absence of perceptual input or rehearsal. The latter, instead, is responsible for representing the characteristics of a familiar environment in an orientation-dependent manner. Both subsystems are based on an intrinsic reference system (e.g., Shelton and McNamara, 2001).

Researchers taking into account the role played by the reference systems in several actions hypothesize that they may form specialized cognitive mechanisms that rely on specific neural networks. In particular, it appears that the hippocampus and the medial temporal lobe provide allocentric environmental representations, while the parietal lobe provides egocentric representations, and the retrosplenial cortex and parieto-occipital sulcus allow both types of representation to interact (for a review see, Burgess, 2008).

From a neural point of view, several studies have shown that egocentric navigation is sub-served by a set of areas related to landmark knowledge (i.e., the parahippocampal place area, Epstein and Ward, 2010), egocentric spatial representation by the parietal cortex (i.e., precuneus and cuneus, inferior parietal lobe) and heading information by the retrosplenial cortex. Instead, allocentric navigation seems mainly related to the hippocampal cortex (Tolman, 1948; O'Keefe and Nadel, 1978; Maguire et al., 1998). Moreover, fMRI studies have shown activations in the hippocampal formation, parietal cortex and retrosplenial regions during tasks involving both egocentric (Galati et al., 2000; Wolber et al., 2004; Latini-Corazzini et al., 2010) and allocentric (Iaria et al., 2007; Latini-Corazzini et al., 2010) frames of reference.

In addition, an ALE meta-analysis by Boccia et al. (2014) demonstrated that the allocentric and egocentric frames of reference were subtended by the same areas, but the latter elicits greater activation in the right precuneus, middle occipital lobe, and angular gyrus.

Studies that investigate the use of egocentric and allocentric frames of reference assume that they are determined mainly by innate factors such as gender (e.g., Chai and Jacobs, 2010), age (e.g., Moffat and Resnick, 2002), or familiarity (e.g., Nori and Piccardi, 2011, 2012). Other studies have considered that the egocentric and allocentric frames of reference may also be affected by external factors such as stimulus salience (e.g., Wolbers and Hegarty, 2010), the availability of landmarks or the experience with these stimuli (Wolbers and Hegarty, 2010). Furthermore, the type of navigational tasks elicits the use of one system rather than the other. For example, route learning tasks elicit egocentric coordinates (e.g., Nemmi et al., 2013), while place learning tasks elicit allocentric ones (e.g., Woolley et al., 2010). However, some studies have revealed that people are able to switch between the two different frames of reference (e.g., Iaria et al., 2003; Etchamendy and Bohbot, 2007; Iglói et al., 2009). Recently, Livingstone-Lee et al. (2014) have studied the effects of performing allocentric and egocentric training in men and women for a subsequent spatial task in which the use of both allocentric and egocentric frames of reference were equally efficient. The results showed no evidence of gender differences in the use of egocentric/allocentric coordinates and in navigation performance, suggesting that individuals may be trained to use one system rather than another. Piccardi et al. (2011a) showed that gender differences in the use of egocentric and allocentric references emerge only in adverse learning conditions when the task requires high spatial skills.

Altogether these studies raise the important issue of needing to deepen our knowledge on what factors might influence the use of ego/allocentric coordinates.

There is also evidence that memories for pathways are viewpoint dependent. As spatial information is encoded according to an orientation-dependent view, the original learning perspective constitutes the primary frame of reference irrespective of the use of egocentric or allocentric coordinates (e.g., Presson and Montello, 1994; Sholl and Nolin, 1997; Shelton and McNamara, 2001). As Piccardi et al. (2011a) pointed out, there is little agreement about which factors are important in attenuating or eliminating orientation-dependent path representations: some studies highlight the environmental characteristics of the path that people have to acquire and remember (e.g., Sholl and Nolin, 1997) whereas others focus on the strategy used for acquiring spatial information (e.g., Rossano et al., 1995; Nori et al., 2006). Piccardi et al. (2011a) observed that when people have the possibility to learn a path without a time limit, basing their strategies on egocentric coordinates, both men and women are good at performing directional judgments irrespective of the learning orientation. In this work, the authors investigated the ability of 106 (55 males, 51 females) college students to recall an 8-step path from different viewpoints after moving directly on the path or by studying the same layout printed on a map. Participants did not have any time limit during the Learning phase. For each participant, authors computed the time and the number of repetitions necessary to learn the path.

Results showed that by allowing longer duration of familiarization and more practice repetitions for females than males markedly reduced the gender difference (for details see Piccardi et al., 2011a). For such a reason, in the present study we have set a time limit corresponding to 3 min and 1 s for men and 3 min and 30 s for women and a number of repetitions corresponding to three times for men and four times for women, adopting the same means and standard deviations obtained by participants in Piccardi et al. (2011a).

This is true also during navigation in virtual environments (Nori et al., 2015a,b).

The aim of the present study was to investigate the presence of gender differences in learning a pathway in different frames of reference (ego/allocentric) and in translating from one frame of reference to another, considering the different time of familiarization for men and women. We also hypothesize that women perform better and more quickly in egocentric as opposed to allocentric frames of reference. Specifically, we hypothesized that adopting the same frame of reference assumed during the learning phase also in the retrieval phase may make participants more accurate and faster in recalling the previously learnt pathway. Vice versa, translating spatial information learnt in one frame of reference to another may affect the accuracy and speediness in the recalling of the learnt path. With respect to gender, we also expected that the translating computation from one frame of reference to another would be more difficult for women than men. A secondary aim of this work was to analyze if increasing the familiarity with the experimental setting (acting through the exposure time to the map or the number of repetitions of the path in the real environment) may produce an improvement in the performance making it easier to translate from one frame of reference to another.

## Method

### Participants

The study involved 160 College students (83 women), recruited in the Sapienza University of Rome, aged between 18 and 30 years (*M* = 24.91 years *S.D*. = 2.41 years; Men, *M* = 25.76 years, *S.D*. = 2.20 years; Women, *M* = 24.03 years, *S.D*. = 2.30 years). Thirteen participants were left-handed and eight ambidextrous (Salmaso and Longoni, 1983). Participants were randomly assigned to one out of four experimental conditions, namely: egocentric frame of reference condition (EC), 40 participants (20 women); allocentric frame of reference condition (AC), 41 participants (21 women); egocentric/allocentric frames of reference condition (EAC), 42 participants (22 women) and allocentric/egocentric frames of reference condition (AEC), 37 participants (20 women). This study was carried out in agreement with the Declaration of Helsinki with written informed consent from all subjects. The protocol was approved by the Local Ethical Committee of the Sapienza University.

## Apparatus and procedure

### Condition 1. EC

#### Egocentric learning phase

**Figure 1:**
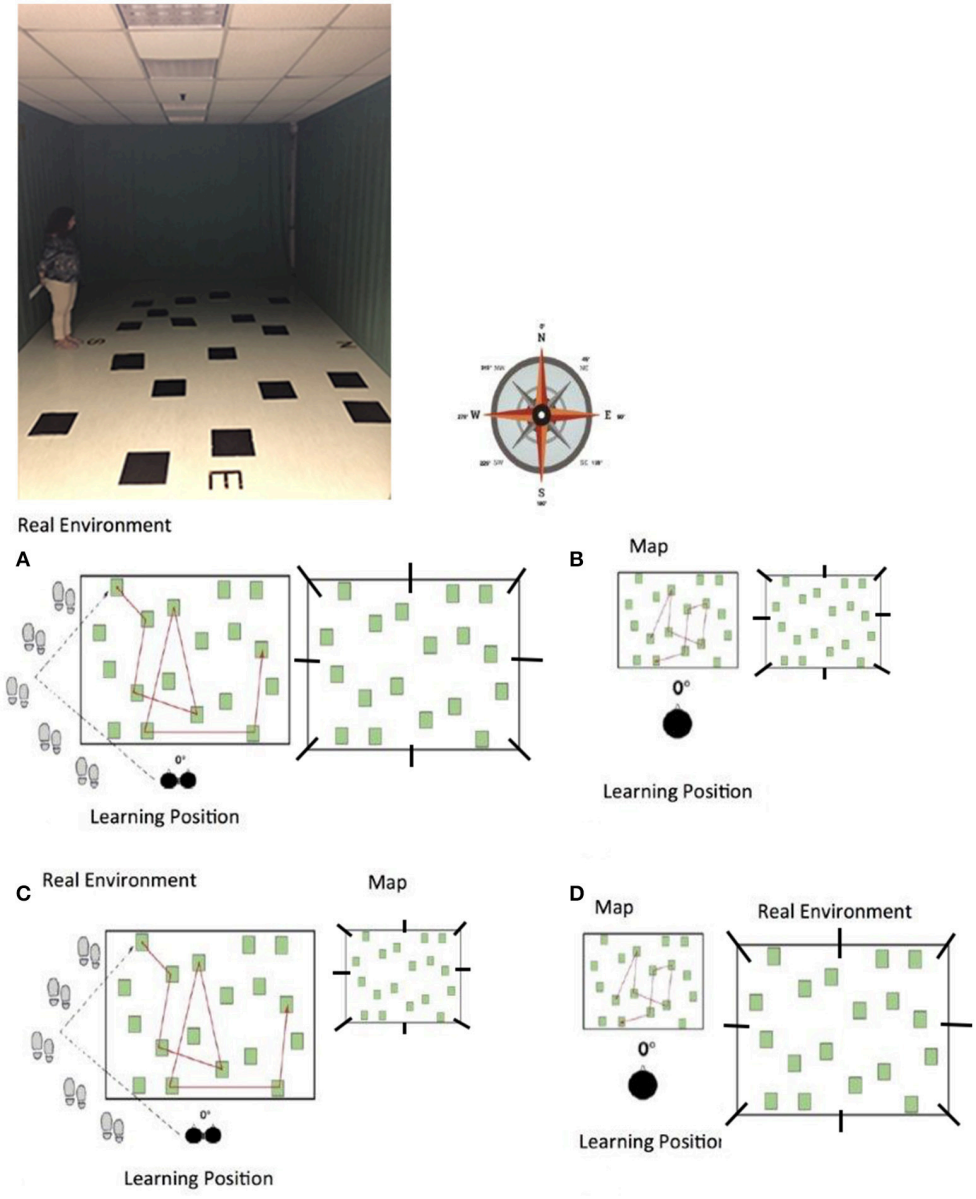
The photo of experimental apparatus and the different experimental condition with the different angle degree where to reproduce the path: EC **(A)** egocentric frame of reference with the same reference system both for learning and retrieval; AC **(B)** allocentric frames of reference with the same reference system both for learning and retrieval; EAC **(C)** different reference system condition, that is to acquire spatial information using an egocentric frame of reference and to retrieve it using allocentric coordinates; AEC **(D)** different reference system condition, that is to acquire spatial information using an allocentric frame of reference and to retrieve it using egocentric coordinates.

Each participant was tested individually. We used an enlarged version of the WalCT (Piccardi et al., 2008, 2013), that is the M-WalCT (used in Piccardi et al., 2011b, 2014, 2016; Nori et al., 2015a,b) in which 18 squares (3 × 3 cm) are placed on a carpet (5 × 6 m) in a scattered array (Figure 1A). To induce route acquisition, the four cardinal points (i.e., North, South, East, West) are indicated outside the carpet. The walls are completely covered with curtains that hide all external landmarks (i.e., doors, heaters, etc.). In this learning condition, participants had to learn four different 8-step sequences. The experimenter demonstrated each sequence by walking on the carpet and stopping on each square for 2 s. On the basis of the findings of Piccardi et al. (2008, 2011a,b), which showed that women require more time to acquire a path than men, and in accordance with Piccardi et al. (2011b, 2014), we considered different times of learning for men and women: the experimenter demonstrated each path three times for men and four times for women. Blindfolded participants were seated in a wheelchair located at the end of the room and were then wheeled, unblindfolded, toward the path. Each participant was then asked to stand up and was taken to the beginning of the path where s/he was led by the experimenter along eight different squares. At the end of each path, the participant was re-seated in the wheelchair and wheeled in a random and meandering route back to the initial location for his/her next walk along the same path, until it had been followed three times for men and four for women. Each walk took ~40 s. Each participant learned four paths and was tested in eight different angle degrees for each path for a total of 32 trails.

#### Egocentric testing phase

After the learning phase, the participants were pushed in the wheelchair again and, unblindfolded, placed in front of the path and were asked to reproduce the path they had learned before in the same or in different perspectives (0°–45°–90°–135°–180°–225°–270°–315°) by walking on the layout. Specifically, the participant was placed in front of the layout in the which s/he had to reproduce the path. The order of the different degrees of reproduction was determined randomly for each path and then the same order was used for all participants (Nori et al., 2006). For each path, the experimenter recorded the points/locations on the path correctly reproduced, that is points/locations in the exact sequence order. A hand-held stopwatch was used to record the planning time, that is the time elapsed before starting the path that had to be reproduced, and response time, that is the time people took to solve the task, including the planning time.

### Condition 2. AC

#### Allocentric learning phase

Each participant was tested individually. We used a map reproduction printed on an A4 sheet of paper of the M-WalCT used in EC, reproduced in 1:10 scale and the same four different 8-step sequences that the participants had to learn (see Figure 1B). On the basis of Piccardi et al. (2011b, 2014), we considered different times of learning for men and women: the experimenter demonstrated each path for 3 min and 1 s for men and 3 min and 30 s for women. Blindfolded participants were seated in a wheelchair located at the end of the room. They were then wheeled, unblindfolded, to a table where they found the first of four paths printed on the map and studied it for the amount of time established for men and women respectively. At the end of the learning phase on each map, the participant was re-seated in the wheelchair and wheeled in a random and meandering route back to the table. In spite of for the allocentric condition to perform participants' disorientation is useless, we have disoriented participants for making the experimental procedure comparable to that of the egocentric condition. Each participant learned four paths printed on a sheet of paper and was tested in eight different angle degrees for each path for a total of 32 trails.

#### Allocentric testing phase

After the learning phase, the participants were asked to reproduce with a pen the path they had previously learned from the same or different perspectives (0°–45°–90°–135°–180°–225°–270°–315°) on a blank map, that is without the path printed on it. Specifically, the blank map was placed in front of the participant in the perspective from which s/he had to reproduce the path. To learn a path from a map requires an allocentric reference frame because it requires to process the whole path whereby the participant represents and updates his/her position in the environment. In such a way, generally, this kind of task is solved by using a reference system external to the body and anchored in the environment (see for example Klatzky, 1998). In such a reference system, the reference directions or axes are stable with respect to the local environment, that is the relationship among landmarks along a path without being centered on the body (Meilinger et al., 2014). We believe that learning a path from a map satisfies this constrain as a consequence we considered this task part of the allocentric testing phase.

The order of the different degrees of reproduction was determined randomly for each path and then the same order was used for all participants (Nori et al., 2006). For each path, the experimenter recorded the points/locations on the map correctly reproduced. A hand-held stopwatch was used to record the planning time, that is the time elapsed before starting the path that had to be reproduced, and response time, that is the time people took to solve the task, including the planning time.

### Condition 3. EAC

#### Egocentric learning phase

The learning phase was the same as in Condition 1, EC: participants had to learn four different 8-step sequences of the M-WalCT. The experimenter demonstrated each sequence by walking on the carpet and stopping on each square for 2 s (see Figure 1C); the experimenter demonstrated each path three times for men and four times for women. Blindfolded participants were seated in a wheelchair located at the end of the room and were then wheeled toward the path. The participant was then asked to stand up and was led to the beginning of the path, where s/he was led by the experimenter along eight different routes. At the end of each path, the participant was re-seated in the wheelchair and wheeled in a random and meandering route back to the initial location for his/her next walk along the same path, until it had been followed three times for men or four for women. Even in this case, each participant was submitted to a total of 32 trails.

#### Allocentric testing phase

After the learning phase, as in Condition 2 AC, the participants were asked to reproduce the path they had previously learned from the same or different perspectives (0°–45°–90°–135°–180°–225°–270°–315°) on a blank map of the M-WalCT. The blank map was placed in front of the participant in the perspective from which s/he had to reproduce the path. The order of these different degrees of reproduction was determined randomly for each path and then the same order was used for all participants (Nori et al., 2006). For each path, the experimenter recorded the points/locations on the map correctly reproduced. A hand-held stopwatch was used to record the planning time, that is the time elapsed before starting the path that had to be reproduced, and response time, that is the time people took to solve the task, including the planning time.

### Condition 4. AEC

#### Allocentric learning phase

The learning phase was the same as for Condition 2, AC: the experimenter demonstrated each path printed on a map for 3 min and 01 s for men and 3 min and 30 s for women (see Figure 1D). Successively, blindfolded participants were seated in a wheelchair located at the end of the room. They were then wheeled to the table where they found the first of four paths. At the end of the learning phase on each map, the participant was seated in the wheelchair and wheeled in a random and meandering route back to the table. Even in this case, each participant was submitted to a total of 32 trails.

#### Egocentric testing phase

After the learning phase, as in Condition 1, EC: the participants were asked to reproduce the path they had previously learned on a map from the same or different perspectives (0°–45°–90°–135°–180°–225°–270°–315°) by walking on the real environment. Participant was placed in front of the layout in the perspective from which s/he had to reproduce the path. As for the previous conditions, the order of these different degrees of reproduction was determined randomly for each path and then the same order was used for all participants (Nori et al., 2006). For each path, the experimenter recorded the points/locations on the path correctly reproduced. A hand-held stopwatch was used to record the planning time, that is the time elapsed before starting the path that had to be reproduced, and response time, that is the time people took to solve the task, including the planning time.

## Results

We compared the four conditions considering a three-way analysis of variance with mixed designs with two levels of the between-variable “gender” (men/women), four levels of between-variable “condition” (EC – AC – EAC – AEC) and the 8 “degrees” of path reproduction as repeated factor (0°–45°–90°–135°–180°–225°–270°–315°). As dependent variable we considered both the mean number of locations correctly reported (accuracy; minimum score = 0, maximum score = 8) and the response time (s.) for each degree (perspective) on the four paths. Both for accuracy and response time, we handled the data, and we averaged them for each participant. In order to analyse if the assumption of homogeneity of variance is met we performed the Levene test (Levene, 1960) on accuracy [0°: *F*_(1, 158)_ = 3.99, *p* = 0.52; 45°: *F*_(1, 158)_ = 0.05, *p* = 0.81; 90°: *F*_(1, 158)_ = 0.01, *p* = 0.92; 135°: *F*_(1, 158)_ = 0.19, *p* = 0.66; 180°: *F*_(1, 158)_ = 0.03, p =.84; 225°: *F*_(1, 158)_ = 0.06, *p* = 0.80; 270°: *F*_(1, 158)_ = 0.17, *p* = 67; 315°: *F*_(1, 158)_ = 0.24, *p* = 0.62], response time [0°: *F*_(1, 158)_ = 3.09, *p* = 0.08; 45°: *F*_(1, 158)_ = 0.1.29, *p* = 0.25; 90°: *F*_(1, 158)_ = 1.40, *p* = 0.23; 135°: *F*_(1, 158)_ = 0.00, *p* = 0.93; 180°: *F*_(1, 158)_ = 1.10, *p* = 0.29; 225°: *F*_(1, 158)_ = 2.55, *p* = 0.11; 270°: *F*_(1, 158)_ = 1.48, *p* = 0.22; 315°: *F*_(1, 158)_ = 1.78, *p* = 0.18], and planning time [0°: *F*_(1, 158)_ = 3.20, *p* = 0.07; 45°: *F*_(1, 158)_ = 0.1.51, *p* = 0.22; 90°: *F*_(1, 158)_ = 4.71, *p* = 0.03; 135°: *F*_(1, 158)_ = 1.35, *p* = 0.24; 180°: *F*_(1, 158)_ = 1.28, *p* = 0.25; 225°: *F*_(1, 158)_ = 1.20, *p* = 0.27; 270°: *F*_(1, 158)_ = 7.29, *p* = 0.00; 315°: *F*_(1, 158)_ = 7.22, *p* = 0.00]. The results show that the assumption of homogeneity of variance is met for accuracy and response time but not for some perspectives (angles) in planning time so we did not perform any further analysis on this variable.

### Number of locations correctly reported (accuracy)

**Figure 2:**
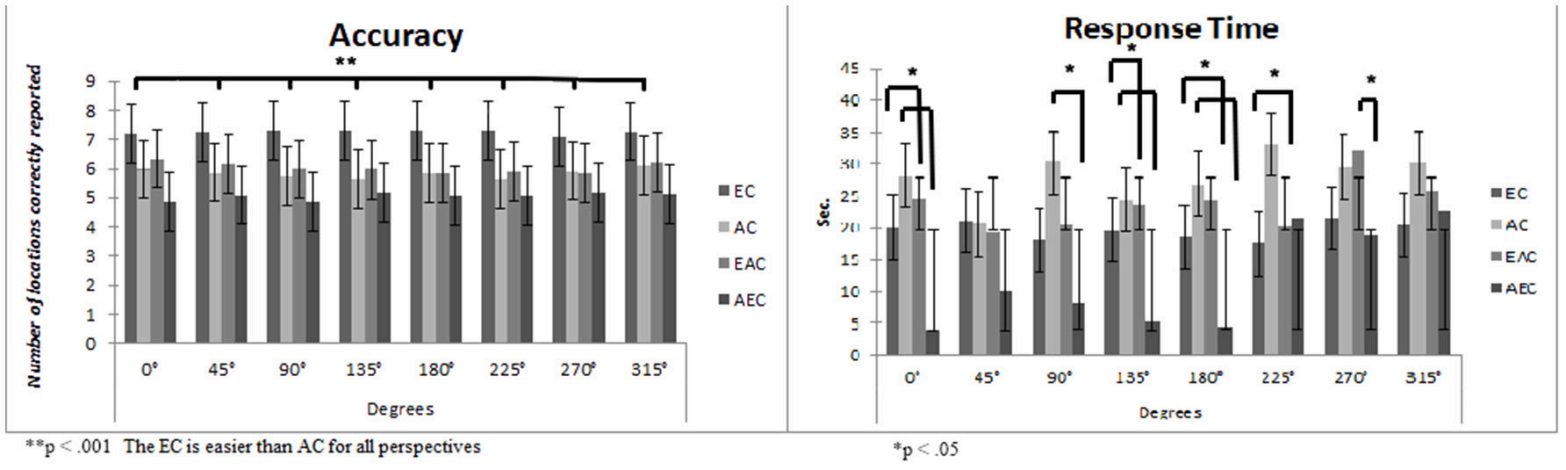
Means and standard deviations considering the four Experimental Conditions (EC, Egocentric Condition; AC, Allocentric Condition; EAC, Egocentric Allocentric Condition; AEC, Allocentric Egocentric Condition) and the eight Degrees in terms of Accuracy and Response Time. The panel on the **right** represents participants' performances in terms of Accuracy, on the y axes is reported the number of location correctly reported (maximum score = 8), while in x axes are reported the degree of reproduction. The panel on the **left** represents participants' performances in terms of Response Time. Specifically, on the y axes is reported the time spent in reproducing the path expressed in seconds, while on the y axes is reported the different degree of path starting.

The main effect of “gender” was not statistically significant [*F*_(1, 152)_ = 0.66, *p* = 0.41, η^2^ = 0.00; Men *M*: 5.93, *S.D*. = 0.19, Women *M* = 6.16, *S.D*. = 0.18], nor was the interaction “gender x condition” [*F*_(3, 152)_ = 1.13, *p* = 0.33, η^2^ = 0.02]. The main effect of “condition” was statistically significant [*F*_(3, 152)_ = 10.77, *p* < 0.001, η^2^ = 0.17]. *Post-hoc* Bonferroni revealed that the EC is easier than all the others (*p*_s_ < 0.01: EC, *M* = 7.25 *S.D*. = 0.27; AC, *M* = 5.85 *S.D*. = 0.26; EAC, *M* = 6.03 *S.D*. = 0.26; AEC, *M* = 5.05 *S.D*. = 0.28). The main effect of “degrees” was statistically significant [*F*_(7, 152)_ = 2.17, *p* = 0.01, η^2^ = 0.01]. *Post-hoc* Bonferroni revealed that it is easier to remember a path from 315° (*M* = 6.18, *S.D*. = 0.37) than from 90° (*M* = 5.97, *S.D*. = 0.14) and 225° (*M* = 5.99, *S.D*. =.14). No other significant results were found. The interaction “condition x degrees” was statistically significant [*F*_(21, 152)_ = 2.84, *p* < 0.001, η^2^ = 0.17]. Specifically, *post-hoc* Bonferroni revealed that remembering a path in a real congruent condition (EC) is easier than in a map/real incongruent condition (AEC) for all perspectives (*p*_s_ < 0.001). Moreover, remembering a path in a real congruent condition (EC) is easier than in a map congruent condition (AC) from 135° and 225° perspectives (see Figure 2). No other significant differences were revealed.

### Response time

The main effect of “gender” was statistically significant [*F*_(1, 152)_ = 4.25, *p* < 0.05, η^2^ = 0.02], men are faster than women (men, *M* = 19.96s., *S.D*. = 0.1.30s.; women, *M* = 22.70s., *S.D*. = 0.81.25s.). Moreover, the main effect of “condition” was also statistically significant [*F*_(3, 152)_ = 13.68, *p* < 0.001, η^2^ = 0.21]. *Post-hoc* Bonferroni showed that remembering a path in AC (*M* = 27.90s., *S.D*. = 1.78s.) was slower than in AEC (*M* = 11.97s, *S.D*. = 1.88s.) and EC (*M* = 19.63s., *S.D*. = 1.80s.). Moreover, the AEC is faster than EC and EAC (*M* = 23.82s., *S.D*. = 1.76s.). The main effect of “degrees” was statistically significant [*F*_(7, 152)_ = 18.71, *p* < 0.001, η^2^ = 0.11]: recalling a path from 225°-270°-315° is slower than all other degrees (*p* < 0.05).

The interaction “condition x degrees” was also statistically significant [*F*_(21, 152)_ = 8.80, *p* < 0.001, η^2^ = 0.14]. Specifically, *post-hoc* Bonferroni showed that remembering a path in the AEC is faster than in AC at 0°–90°–135°–180° (*p*_s_ < 0.01); remembering a path in AC is slower than in EC and EAC at 225° (*p*_s_ < 0.05) while it is faster than in EC and in EAC at 0°–135°–180° (*p*_s_ < 0.05); remembering a path in AEC is faster than in EAC at 270° (*p* < 0.05). Descriptives are shown in Figure 2. No other significant differences are shown.

## Discussion

To our knowledge, the present study was the first to investigate the presence of gender differences in retrieving environmental information learnt in the same or different frames of reference. In particular, our intent was to analyse whether the performance of men and women in retrieving a path from different points of view using the same reference both in the learning and retrieval phases is easier and faster than one in which different references are used. We also wanted to investigate whether gender differences could be reduced by increasing the familiarity with the experimental setting by manipulating the time of map exposure or the number of repetitions to learn a pathway.

Our results partially confirm our hypothesis. Gender differences in remembering a path correctly were absent in both egocentric and allocentric reference frames. In particular, we found that by manipulating familiarity, gender differences disappeared or attenuated. This result differs from that of several studies in which men outperform women in perspective-taking tasks (e.g., for a review see Coluccia and Louse, 2004). However, in these studies men and women were exposed to the same short time to acquire spatial information. Already, Piccardi et al. (2011a,b) found that computing the individual learning time allows gender differences in perspective taking tasks to be eliminated. Therefore, also computational spatial operations like the transformation of spatial information from egocentric to allocentric frames of reference and vice versa do not elicit a gender effect. This supports the evidence that when spatial information is stored and consolidated, all individuals are able to process and re-elaborate it. This result is in line with studies which demonstrated that by increasing the familiarity with the environment even poor spatial navigators were able to perform very complex tasks: the higher the individuals' familiarity with the environment, the better their performances (Iachini et al., 2009; Nori and Piccardi, 2011; Piccardi et al., 2011a). Indeed, as pointed out by Montello (1998) additional locomotor and perceptual experience of the environment, as well as familiarity with the place, result in more extensive, complete, and accurate knowledge. So the two concepts are related and specifically, increasing learning time increases familiarity with the environment, thus allowing a more complete knowledge of the experimental environment. Our results are also in line with Iachini et al. (2009), who investigated how familiarity and gender have an effect even in the frames of reference used in memory to represent a real environment. Their results showed that males were more accurate and faster than females in detecting environmental changes, in particular when participants were unfamiliar with the environment. Considering that in our experiment, the degree of familiarity with the environment was measured in terms of the time necessary to learn the experimental array, we found that when time and repetitions are sufficient, women are as efficient as men. However, it is interesting that gender differences are still present in solving the task: men are quicker than women. Therefore, familiarity is important to eliminate gender differences in terms of accuracy but it is not sufficient to eliminate gender differences in response time. This result could be explained by considering the model of Coluccia and Louse (2004), who proposed that gender differences emerged according to the cognitive demand of the spatial tasks, which could be attributed to the visuo-spatial working memory load. Specifically, gender differences emerged only when tasks required a high integration and transformation of visually imagined material: given the necessary time to acquire spatial information females may rotate the path accurately even if they take longer than males to do so, since they are accurate but still slow in performing the mental rotation of the environment.

Another result that emerged from our study is that all participants showed the same level of accuracy in all perspectives they assumed to recall the path. In general, all preferred clockwise and right body axes starting positions with respect to counter clockwise and left body axes positions, probably because the participants were right-handed (see also: Sholl and Nolin, 1997). The time of response is predicted by the time employed to mentally process the cognitive map of the environment. In particular, we found that the planning time predicted some perspectives in terms of time of performance.

As far as the effect of learning and retrieval spatial information is concerned, using the same reference frame, we found that the egocentric frame of reference condition (EC) is the easiest for all participants in recalling a path from different perspectives, while the allocentric frame of reference condition (AC) resulted in a worse performance. This is in line with Wang and Spelke's model (Wang and Spelke, 2000, 2002), which suggests that the egocentric coordinates are the preferred system for both men and women, even if the use of allocentric coordinates could be not completely excluded, and it is possible that participants may simultaneously use an allocentric reference in which they themselves are the references for the spatial information (Burgess, 2006). Very likely, men are more proficient than women in adverse learning conditions (e.g., temporal pressure or when the task requires a high cognitive load) (Coluccia and Louse, 2004; Lawton, 2010). Generally, in fact, during a complex environment exploration requiring a prolonged self-motion, it is more efficient to maintain an allocentric map of the environment than to continuously update multiple egocentric representations. However, the translation between egocentric and allocentric information is not equal: it is much more difficult to translate from egocentric to allocentric information than vice versa. The translation between these two different systems of coordinates that requires translating action-oriented egocentric representations into allocentric representations (e.g., the body references right/left become environmental references north/south) determines a cost in terms of time (Iaria et al., 2003; Etchamendy and Bohbot, 2007; Iglói et al., 2009). However, data demonstrate that individuals are nevertheless able to switch from one representation to another also during navigation, suggesting that people have both egocentric and allocentric coordinates at their disposal, although it is generally agreed that allocentric representations are more cognitive demanding than egocentric ones. This is also supported by Siegel and White's model (1975) that suggests a cumulative and hierarchical organization of spatial knowledge with high-level stages encompassing features of the lower stages. For these authors, in order to have an allocentric representation of the environment it is necessary to have acquired the egocentric representation. This seminal model supports our results that demonstrate how learning and retrieving a path is easier through an egocentric frame of reference. Indeed, from this point of view, the egocentric reference is the first system that humans develop to move through the environment. The importance of starting from an egocentric first-person perspective representation is also assumed in the animal models of spatial navigation (O'Keefe and Nadel, 1979). Egocentric navigation, in fact, resembles Taxon navigation (O'Keefe and Nadel, 1979), whereas allocentric navigation resembles Place navigation (O'Keefe and Nadel, 1979). Moreover, Byrne and Becker (2008) focus on the fact that spatial information from the environment must reach the brain via sensory receptors in an inherently egocentric sensory representation.

To summarize, gender differences in spatial navigation performance are reduced when participants become familiar with the environment. In our case, the familiarity was acquired through the time of exposure to the map or the number of repetitions performed to learn the path. With a high level of familiarity participants are able to perform spatial tasks with a high cognitive load, such as the translating from one frame of reference to another. In general, participants show a preference and a better proficiency when learning occurs in egocentric frames of reference. Overall, these results indicate that interactions between environmental demands and cognitive processes compensate for gender differences in spatial navigation performance.

## Author contributions

RN and LP: made substantial contributions to conception and design, participated in drafting the article, or revising it critically for important intellectual content; AM, MG, AR, and OA: made contributions to acquisition of data, and analysis and interpretation of data; CG: gave contributions in interpretation of data; final approval of the version to be submitted and any revised version.

### Conflict of interest statement

The authors declare that the research was conducted in the absence of any commercial or financial relationships that could be construed as a potential conflict of interest.
